# The genome sequence of the pine hoverfly, *Blera fallax* (Linnaeus, 1758)

**DOI:** 10.12688/wellcomeopenres.19034.1

**Published:** 2023-02-20

**Authors:** Helen R. Taylor, Heather Ritchie-Parker, Wellcome Sanger Institute Tree of Life programme, Wellcome Sanger Institute Scientific Operations: DNA Pipelines collective, Tree of Life Core Informatics collective, Darwin Tree of Life Consortium

**Affiliations:** 1 Field Conservation, Royal Zoological Society of Scotland, Edinburgh, Scotland, UK; 2 WildGenes Laboratory, Royal Zoological Society of Scotland, Edinburgh, Scotland, UK

**Keywords:** Blera fallax, pine hoverfly, genome sequence, chromosomal, Diptera

## Abstract

We present a genome assembly from an individual male *Blera fallax* (the pine hoverfly; Arthropoda; Insecta; Diptera; Syrphidae). The genome sequence is 462 megabases in span. Most of the assembly is scaffolded into 7 chromosomal pseudomolecules, including the assembled X and Y chromosome. The mitochondrial genome has also been assembled, and is 16.2 kilobases in length.

## Species taxonomy

Eukaryota; Metazoa; Ecdysozoa; Arthropoda; Hexapoda; Insecta; Pterygota; Neoptera; Endopterygota; Diptera; Brachycera; Muscomorpha; Syrphoidea; Syrphidae; Eristalinae; Xylotini; *Blera*; *Blera fallax* (Linnaeus, 1758) (NCBI:txid226147).

## Background

**Figure 1.**
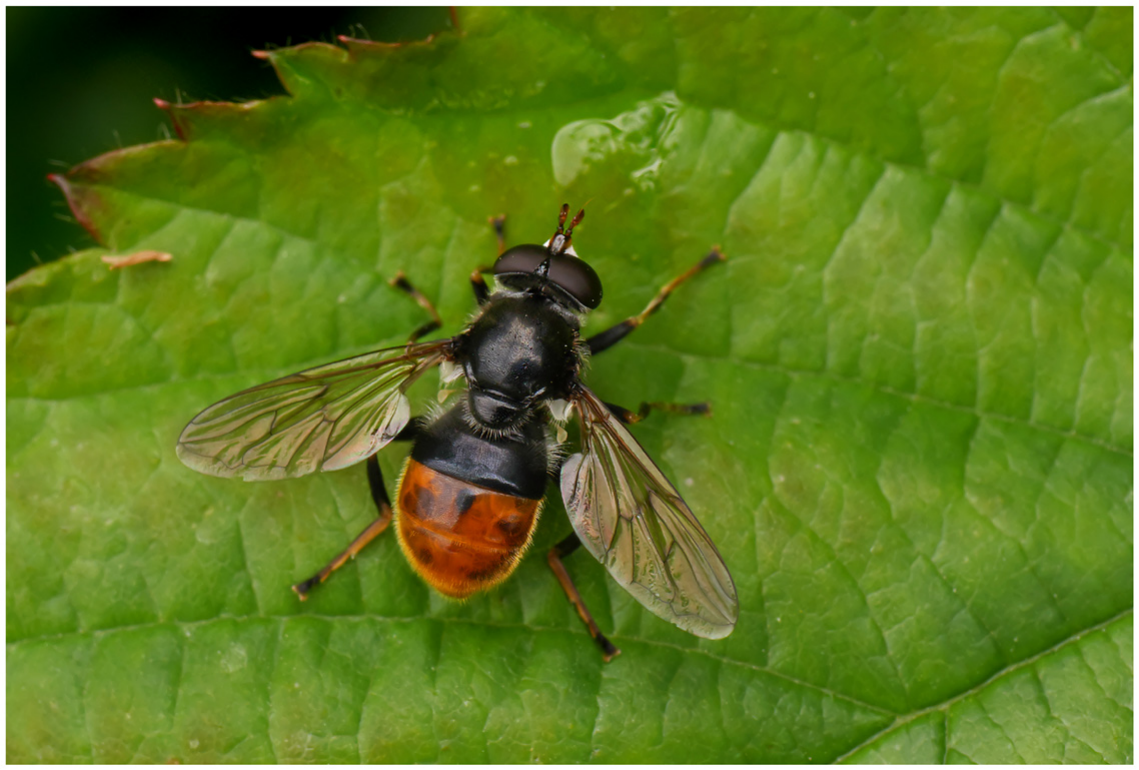
Photograph of *Blera fallax*. Photograph by Franck Vassen (CC-By 2.0).

The pine hoverfly ( *Blera fallax*) ( Figure 1) is a member of the Syrphidae and is one of Britain’s rarest invertebrates. Although found across the palearctic, in Britain this species is restricted to just one small forest patch in the Cairngorms National Park and is listed as Critically Endangered in the UK Red Data Book ( Ball & Morris, 2015; Taylor *et al.*, 2021). Their decline is linked to habitat loss; pine hoverflies require diverse, old growth Caledonian pine forests to support their various life stages. In Britain, much of this habitat has been lost to deforestation, and land use change ( Rotheray & MacGowan, 2000).

Intensive conservation efforts for the pine hoverfly are currently underway via the Rare Invertebrates in the Cairngorms (RIC) partnership. As part of this project, a conservation breeding programme for the species was initiated at the Royal Zoological Society of Scotland’s Highland Wildlife Park in 2018. Alongside the breeding programme, habitat creation has been undertaken at RSPB and Forestry and Land Scotland-owned sites over the past five years by other RIC partners. In 2021/22, around 6,000 pine hoverfly larvae from the breeding programme were released across three wild sites where habitat creation had taken place, with the aim of boosting the number of populations of this species in Scotland. Breeding in these released populations was confirmed in September 2022 and further top-up releases are underway.

Only one study, to date, has examined the genetic diversity of pine hoverflies in Britain ( Rotheray *et al.*, 2012b) and genetic resources for the species are limited ( Rotheray *et al.*, 2012a). Understanding what genetic diversity is present in the wild following the sharp decline of pine hoverflies over the past few decades is important for understanding the long-term genetic viability of the species. The conservation breeding population for pine hoverflies was founded with eggs from just two females, and it is unclear how much of the native genetic diversity has been captured and maintained within the breeding programme. Furthermore, if the current reintroduction efforts fail, a potential alternative is to reintroduce the species using animals from Scandinavia, but the genomic differences between British and Scandinavian populations have not been established ( Taylor *et al.*, 2021) (although see Rotheray *et al.*, 2012b). This genome will provide a reference to help answer these and other questions, improving the conservation management of pine hoverflies and their long-term prospects in Britain.

## Genome sequence report

The genome was sequenced from one male *B. fallax* collected from Highland Wildlife Park, Scotland, UK (latitude 57.11, longitude –3.97). A total of 56-fold coverage in Pacific Biosciences single-molecule HiFi long reads was generated. Primary assembly contigs were scaffolded with chromosome conformation Hi-C data. Manual assembly curation corrected 25 missing joins or mis-joins, reducing the scaffold number by 11.43% and increasing the scaffold N50 by 3.01%.

**Figure 2.**
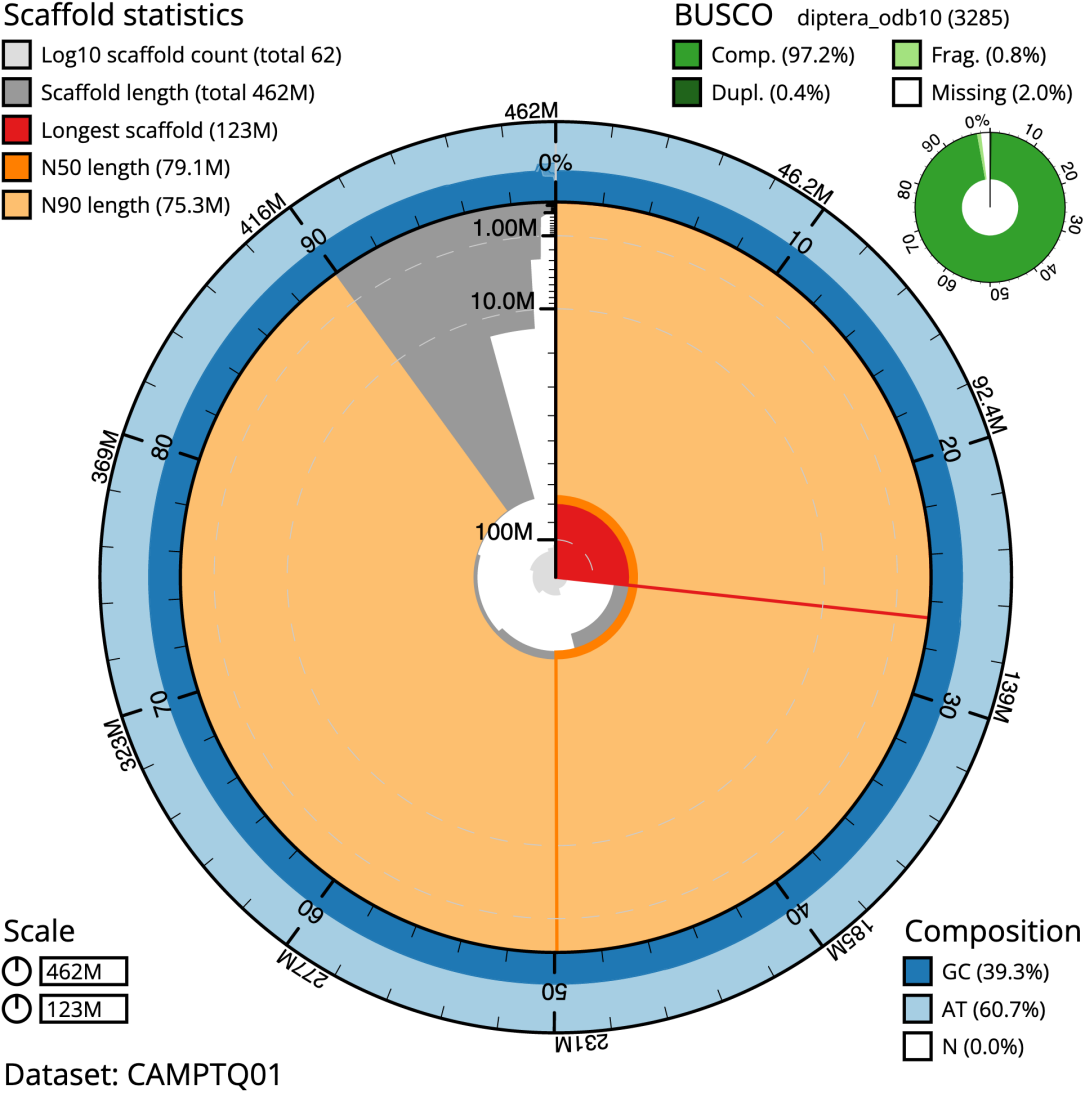
Genome assembly of *Blera fallax*, idBleFall4.1: metrics. The BlobToolKit Snailplot shows N50 metrics and BUSCO gene completeness. The main plot is divided into 1,000 size-ordered bins around the circumference with each bin representing 0.1% of the 461,837,650 bp assembly. The distribution of scaffold lengths is shown in dark grey with the plot radius scaled to the longest scaffold present in the assembly (123,392,791 bp, shown in red). Orange and pale-orange arcs show the N50 and N90 scaffold lengths (79,067,313 and 75,264,518 bp), respectively. The pale grey spiral shows the cumulative scaffold count on a log scale with white scale lines showing successive orders of magnitude. The blue and pale-blue area around the outside of the plot shows the distribution of GC, AT and N percentages in the same bins as the inner plot. A summary of complete, fragmented, duplicated and missing BUSCO genes in the diptera_odb10 set is shown in the top right. An interactive version of this figure is available at https://blobtoolkit.genomehubs.org/view/idBleFall4.1/dataset/CAMPTQ01/snail.

The final assembly has a total length of 461.8 Mb in 62 sequence scaffolds with a scaffold N50 of 79.1 Mb ( Table 1). Most (99.21%) of the assembly sequence was assigned to seven chromosomal-level scaffolds, representing five autosomes and the X and Y sex chromosomes. Chromosome-scale scaffolds confirmed by the Hi-C data are named in order of size. ( Figure 2– Figure 5; Table 2). The assembly has a BUSCO v5.3.2 ( Manni *et al.*, 2021) completeness of 97.2% using the diptera_odb10 reference set. While not fully phased, the assembly deposited is of one haplotype. Contigs corresponding to the second haplotype have also been deposited.

**Table 1.** Genome data for *Blera fallax*, idBleFall4.1.

Project accession data		
Assembly identifier	idBleFall4.1	
Species	*Blera fallax*	
Specimen	idBleFall4	
NCBI taxonomy ID	226147	
BioProject	PRJEB55743	
BioSample ID	SAMEA9654431	
Isolate information	male; idBleFall4 (PacBio) male; idBleFall1 (Hi-C)	
Assembly metrics *		*Benchmark*
Consensus quality (QV)	66.4	*≥ 50*
*k*-mer completeness	100%	*≥ 95%*
BUSCO **	C:97.2%[S:96.8%,D:0.4%], F:0.8%,M:2.0%,n:3,285	*C ≥ 95%*
Percentage of assembly mapped to chromosomes	99.21%	*≥ 95%*
Sex chromosomes	X and Y chromosomes	*localised homologous pairs*
Organelles	Mitochondrial genome assembled	*complete single alleles*
Raw data accessions		
PacificBiosciences SEQUEL II	ERR10168731	
Hi-C Illumina	ERR10149560	
Genome assembly		
Assembly accession	GCA_946965025.1	
*Accession of alternate haplotype*	GCA_946965035.1	
Span (Mb)	461.8	
Number of contigs	185	
Contig N50 length (Mb)	5.6	
Number of scaffolds	62	
Scaffold N50 length (Mb)	79.1	
Longest scaffold (Mb)	123.4	

* Assembly metric benchmarks are adapted from column VGP-2020 of “Table 1: Proposed standards and metrics for defining genome assembly quality” from ( Rhie *et al.*, 2021). ** BUSCO scores based on the diptera_odb10 BUSCO set using v5.3.2. C = complete [S = single copy, D = duplicated], F = fragmented, M = missing, n = number of orthologues in comparison. A full set of BUSCO scores is available at https://blobtoolkit.genomehubs.org/view/idBleFall4.1/dataset/CAMPTQ01/busco.

**Figure 3.**
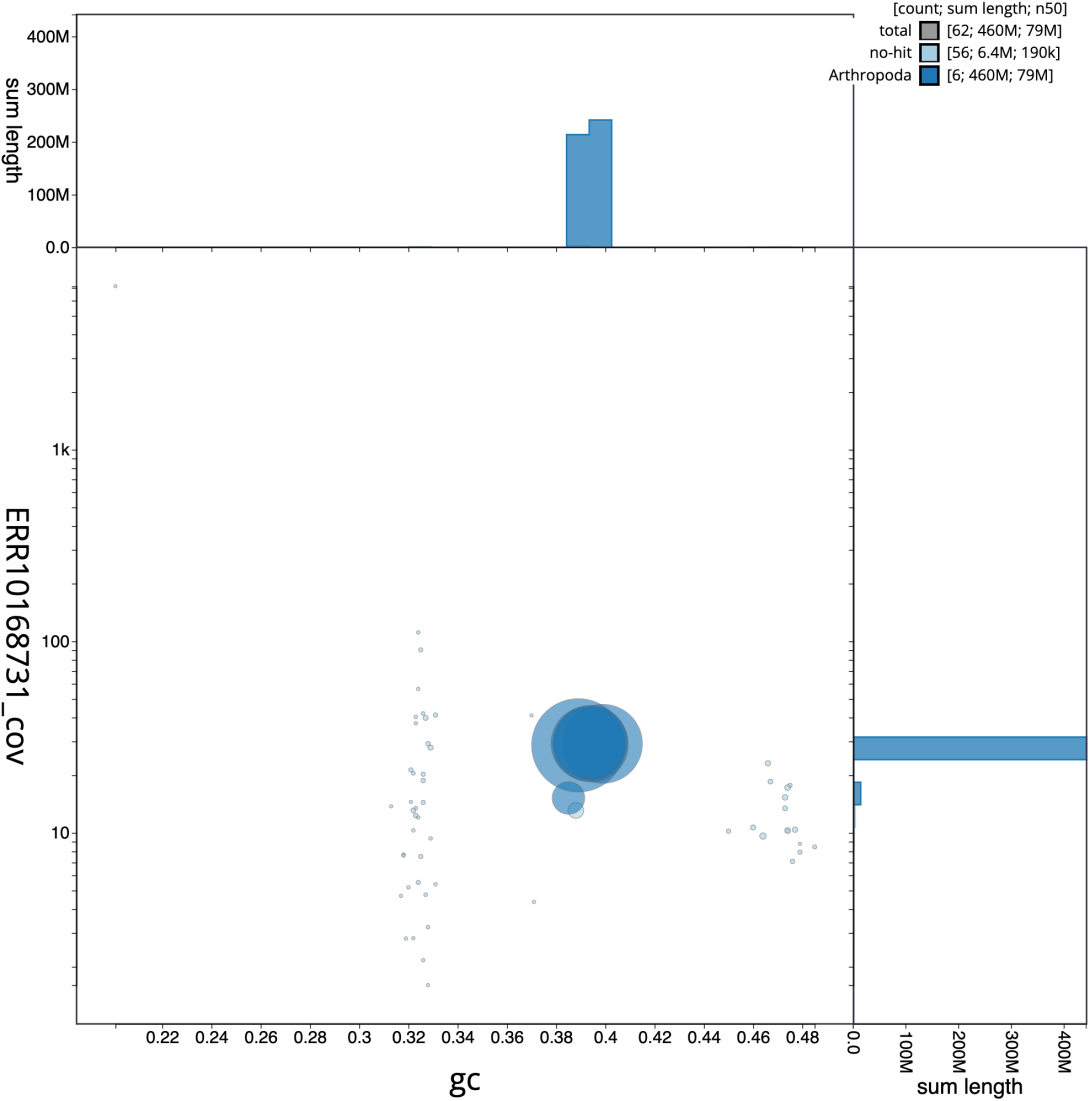
Genome assembly of *Blera fallax*, idBleFall4.1: GC coverage. BlobToolKit GC-coverage plot. Scaffolds are coloured by phylum. Circles are sized in proportion to scaffold length. Histograms show the distribution of scaffold length sum along each axis. An interactive version of this figure is available at https://blobtoolkit.genomehubs.org/view/idBleFall4.1/dataset/CAMPTQ01/blob.

**Figure 4.**
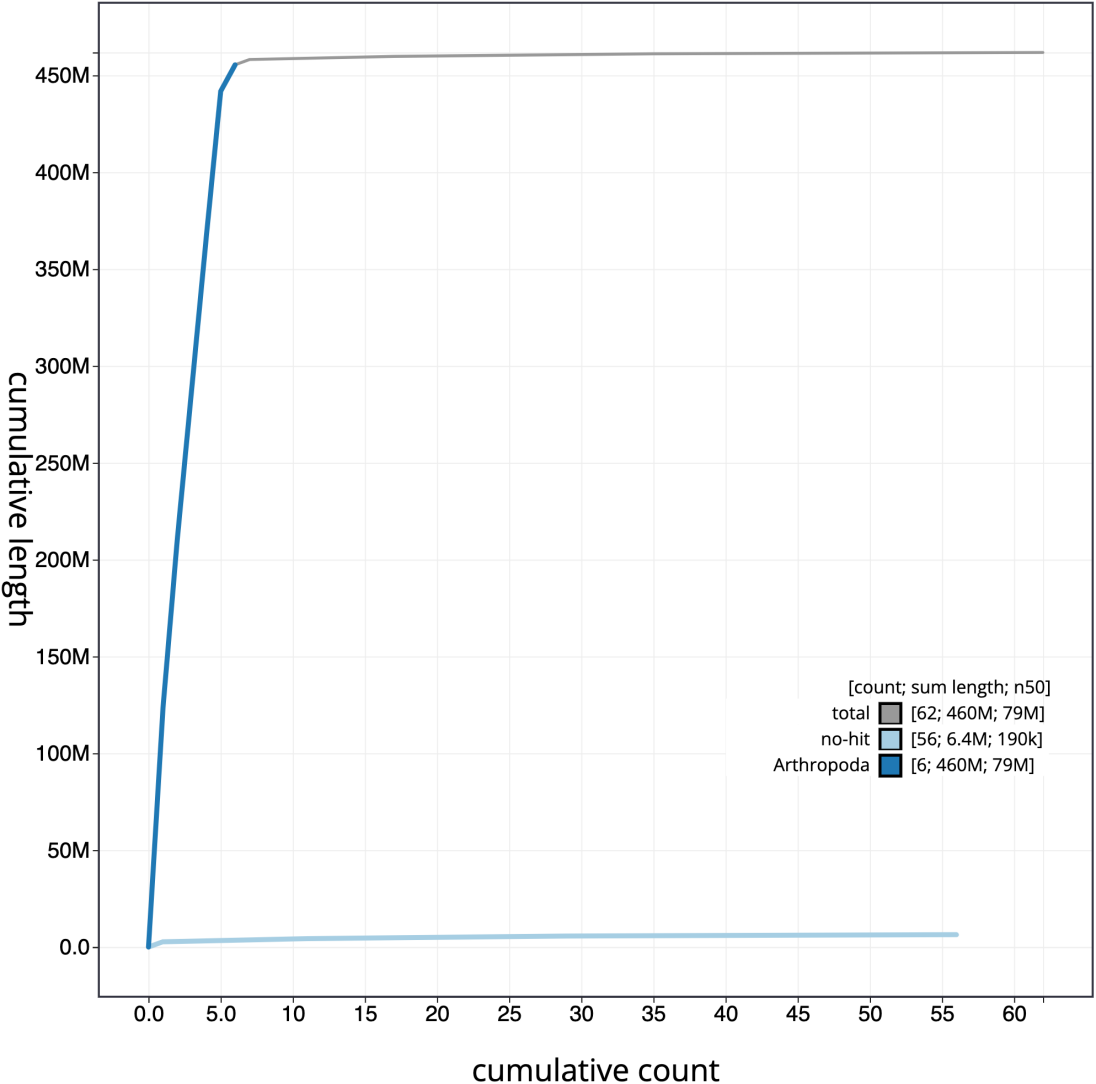
Genome assembly of *Blera fallax*, idBleFall4.1: cumulative sequence. BlobToolKit cumulative sequence plot. The grey line shows cumulative length for all scaffolds. Coloured lines show cumulative lengths of scaffolds assigned to each phylum using the buscogenes taxrule. An interactive version of this figure is available at https://blobtoolkit.genomehubs.org/view/idBleFall4.1/dataset/CAMPTQ01/cumulative.

**Figure 5.**
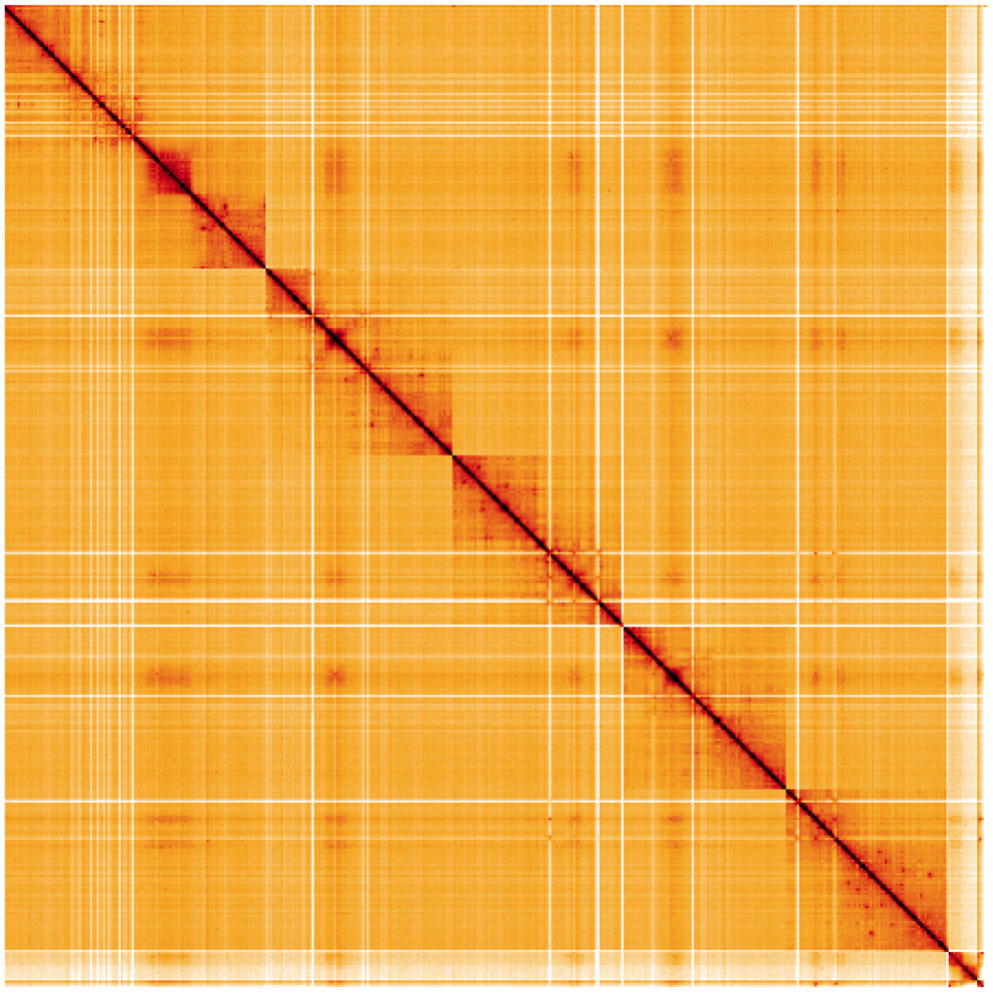
Genome assembly of *Blera fallax*, idBleFall4.1: Hi-C contact map. Hi-C contact map of the idBleFall4.1 assembly, visualised using HiGlass. Chromosomes are shown in order of size from left to right and top to bottom. An interactive version of this figure may be viewed at https://genome-note-higlass.tol.sanger.ac.uk/l/?d=SepYMnr5SZ-Z_46DLU-b_w.

**Table 2.** Chromosomal pseudomolecules in the genome assembly of *Blera fallax*, idBleFall4.

INSDC accession	Chromosome	Size (Mb)	GC%
OX337244.1	1	123.39	38.9
OX337245.1	2	87.33	39.9
OX337246.1	3	79.07	39.4
OX337247.1	4	76.75	39.3
OX337248.1	5	75.26	39.4
OX337249.1	X	13.66	38.5
OX337250.1	Y	2.73	38.8
OX337251.1	MT	0.02	20.4

## Methods

### Sample acquisition and nucleic acid extraction

Two *B. fallax* specimens (idBleFall1 and idBleFall4) were collected from Highland Wildlife Park, Scotland, UK (latitude 57.11, longitude –3.97) in June 2021. The specimens were collected and identified by Helen Taylor (Royal Zoological Society of Scotland). The idBleFall4 specimen used for DNA sequencing was placed in a liquid nitrogen dry shipper immediately after death.

DNA was extracted from whole organism tissue of idBleFall4 at the Wellcome Sanger Institute (WSI) Scientific Operations core from the whole organism using the Qiagen MagAttract HMW DNA kit, according to the manufacturer’s instructions.

### Sequencing

Pacific Biosciences HiFi circular consensus and 10X Genomics read cloud DNA sequencing libraries were constructed according to the manufacturers’ instructions. DNA sequencing was performed by the Scientific Operations core at the WSI on Pacific Biosciences SEQUEL II (HiFi) instrument. Hi-C data were also generated from whole tissue of idBleFall1 using the Arima v2 kit and sequenced on the Illumina NovaSeq 6000 instrument.

### Genome assembly

Assembly was carried out with Hifiasm ( Cheng *et al.*, 2021) and haplotypic duplication was identified and removed with purge_dups ( Guan *et al.*, 2020). The assembly was then scaffolded with Hi-C data ( Rao *et al.*, 2014) using YaHS ( Zhou *et al.*, 2023). The assembly was checked for contamination and corrected using the gEVAL system ( Chow *et al.*, 2016) as described previously ( Howe *et al.*, 2021). Manual curation was performed using gEVAL, HiGlass ( Kerpedjiev *et al.*, 2018) and Pretext ( Harry, 2022). The mitochondrial genome was assembled using MitoHiFi ( Uliano-Silva *et al.*, 2022), which performed annotation using MitoFinder ( Allio *et al.*, 2020). The genome was analysed and BUSCO scores generated within the BlobToolKit environment ( Challis *et al.*, 2020). Table 3 contains a list of software tool versions used, where relevant.

**Table 3.** Software tools and versions used.

Software tool	Version	Source
BlobToolKit	3.5.0	Challis *et al.*, 2020
gEVAL	N/A	Chow *et al.*, 2016
Hifiasm	0.16.1-r375	Cheng *et al.*, 2021
HiGlass	1.11.6	Kerpedjiev *et al.*, 2018
MitoHiFi	2	Uliano-Silva *et al.*, 2022
PretextView	0.2	Harry, 2022
purge_dups	1.2.3	Guan *et al.*, 2020
YaHS	yahs-1.1.91eebc2	Zhou *et al.*, 2023

### Ethics and compliance issues

The materials that have contributed to this genome note have been supplied by a Darwin Tree of Life Partner. The submission of materials by a Darwin Tree of Life Partner is subject to the Darwin Tree of Life Project Sampling Code of Practice.. By agreeing with and signing up to the Sampling Code of Practice, the Darwin Tree of Life Partner agrees they will meet the legal and ethical requirements and standards set out within this document in respect of all samples acquired for, and supplied to, the Darwin Tree of Life Project. All efforts are undertaken to minimise the suffering of animals used for sequencing. Each transfer of samples is further undertaken according to a Research Collaboration Agreement or Material Transfer Agreement entered into by the Darwin Tree of Life Partner, Genome Research Limited (operating as the Wellcome Sanger Institute), and in some circumstances other Darwin Tree of Life collaborators.

## Data Availability

European Nucleotide Archive: *Blera fallax* (pine hoverfly). Accession number PRJEB55743; https://identifiers.org/ena.embl/PRJEB55743. ( Wellcome Sanger Institute, 2022) The genome sequence is released openly for reuse. The *Blera fallax* genome sequencing initiative is part of the Darwin Tree of Life (DToL) project. All raw sequence data and the assembly have been deposited in INSDC databases. The genome will be annotated using available RNA-Seq data and presented through the Ensembl pipeline at the European Bioinformatics Institute. Raw data and assembly accession identifiers are reported in Table 1.

## Author information

Members of the Wellcome Sanger Institute Tree of Life programme are listed here: https://doi.org/10.5281/zenodo.4783585.

Members of Wellcome Sanger Institute Scientific Operations: DNA Pipelines collective are listed here: https://doi.org/10.5281/zenodo.4790455.

Members of the Tree of Life Core Informatics collective are listed here: https://doi.org/10.5281/zenodo.5013541.

Members of the Darwin Tree of Life Consortium are listed here: https://doi.org/10.5281/zenodo.4783558.
