# Supplementary material for: The genome sequence of the pine hoverfly, Blera fallax (Linnaeus, 1758)
Source: Wellcome Open Res. 2025 Feb 21;8:89. Originally published 2023 Feb 20. [Version 2] doi: 10.12688/wellcomeopenres.19034.2 (PMC12120433; doi:10.12688/wellcomeopenres.19034.2)
Supplement: Peer review history

# Reviewer report: Reviewer response for version 2

**DOI:** 10.21956/wellcomeopenres.26277.r121876

**Published:** 2025-05-28

**Authors:** Henrique Antoniolli

**Affiliations:** 1 Universidade Federal do Rio Grande do Sul (UFRGS), Porto Alegre, Brazil

In the present study, Taylor et al. report a contiguous genome assembly for a pine hoverfly, *Blera fallax*. The introduction section brings an overview on its biology, along with motivations for sequencing its genome. The methods are well described and align with current literature. All assembly statistics yielded satisfactory results. Therefore I recommend indexing this paper.

Are sufficient details of methods and materials provided to allow replication by others?

Yes

Is the rationale for creating the dataset(s) clearly described?

Yes

Are the datasets clearly presented in a useable and accessible format?

Yes

Are the protocols appropriate and is the work technically sound?

Yes

Reviewer Expertise:

Genomics, evolution

I confirm that I have read this submission and believe that I have an appropriate level of expertise to confirm that it is of an acceptable scientific standard.

# Reviewer report: Reviewer response for version 2

**DOI:** 10.21956/wellcomeopenres.26277.r119997

**Published:** 2025-03-13

**Authors:** Komal Kumar Raja

**Affiliations:** 1 Baylor College of Medicine, Houston, TX, USA

The article by Taylor et al. presents the genome sequence of the pine hoverfly, *Blera fallax*. The authors have provided a detailed introduction focusing on the importance of conservation efforts by the community on this species. The genome assembly metrics are impressive and show that the assembly is high quality with 97.2% BUSCO score. DNA extraction and other experimental methods along with all the bioinformatics tools utilized have been presented in the manuscript, which allow for reproducibility. Overall, all relevant data have been included in the manuscript. It is well written and is a good resource for comparative genomics researchers.

Are sufficient details of methods and materials provided to allow replication by others?

Yes

Is the rationale for creating the dataset(s) clearly described?

Yes

Are the datasets clearly presented in a useable and accessible format?

Yes

Are the protocols appropriate and is the work technically sound?

Yes

Reviewer Expertise:

Single cell genomics, Bioinformatics, genomics

I confirm that I have read this submission and believe that I have an appropriate level of expertise to confirm that it is of an acceptable scientific standard.

# Reviewer report: Reviewer response for version 1

**DOI:** 10.21956/wellcomeopenres.21103.r78350

**Published:** 2024-05-03

**Authors:** Wen-Jun Bu

**Affiliations:** 1 Nankai University, Tianjin, Tianjin, China

Biodiversity conservation is a collective responsibility shared by all of humanity. The authors present a reference genome for the pine hoverfly ( *Blera fallax*), a rare British invertebrate. This genomic resource facilitates the monitoring of genetic diversity within populations and the assessment of genomic differences between geographic groups. These insights hold significant value for population reintroduction and interregional population introductions in conservation efforts.

 Based on the provided assembly statistics, the authors assembled a highly quality genome using PacBio HiFi and Hi-C data. But there are still some concerns with the current version of the article.

 1. It is recommended to provide detailed usage parameters and analysis details in the method section. The current description is too general.

 2. In this article, the identification of X and Y sex chromosomes seems only by size. It is recommended to provide more evidence to identify which scaffolds are sex chromosomes.

 3. According to the methods, 10X genomics read cloud DNA libraries were constructed, however, it is not described in detail whether the data generated from the libraries was used for genome assembly. And I am unable find it through the link provided at the end of the paper.

 4. According to the Table 1, some indicators were used to evaluate the assembled genome, but this section was not clear in methods, for example, the QV value, which software and data are used to evaluate the QV value.

 5. It is difficult to identify the chromosomes boundaries in Figure 5, and the figure lacks of the labelling of chromosomes.

Are sufficient details of methods and materials provided to allow replication by others?

Partly

Is the rationale for creating the dataset(s) clearly described?

Yes

Are the datasets clearly presented in a useable and accessible format?

Partly

Are the protocols appropriate and is the work technically sound?

Yes

Reviewer Expertise:

Insect systematics, population genomics

I confirm that I have read this submission and believe that I have an appropriate level of expertise to confirm that it is of an acceptable scientific standard, however I have significant reservations, as outlined above.

# Reviewer report: Reviewer response for version 1

**DOI:** 10.21956/wellcomeopenres.21103.r58836

**Published:** 2023-07-14

**Authors:** Kalle Tunström

**Affiliations:** 1 Stockholm University, Stockholm, Sweden

The authors provide a new reference genome for the pine hoverfly (blera fallax), a resource that hopefully can and will be used for future genomic investigations and conservation efforts. Considering the species decline due to reduction of habitat, and transplantation efforts, being able to better monitor their genetic diversity and study local adaptation is of great value.

 The authors used an appropriate combination of PacBio HiFi and Hi-C, together with Hifi-asm and YaHS to assemble a highly contiguous with exceptionally high QVscore of 66! Overall I think this looks like a good assembly based on the provided assembly statistics.

 However while I am sure that the genome is mostly solid, I have some major concerns with the report in its current form.

 Critiques:

1. While I appreciate the clearly described list of most tools and version of said tools that were used, I am missing all the options and settings these tools were used with, considering that this genome, and other DTOL genomes are assembled with what I assume is a fairly standard pipeline, generating a report of all settings and options should not be hard, alternatively providing a link to the scripts used would be good. As it is right now I doubt I could reproduce these results precisely.
2. In the Hi-C scaffolding section there is no information about which short-read aligner was used and if the resulting bam files were processed in some way prior to producing the contact map.
3. There is also something up with the data repository link provided at the end of the paper, while it takes you to a valid project id, there are no links to the assemblies them selves or the raw read files within it. I can find links to both on the DTOL data portal, but this takes you to a project with a different project ID ( PRJEB56199). The accession numbers in the data table does appear to match with this other project ID.
4. According to the methods it is stated that two specimen were collected, however when I look at the DTOL data portal it looks like at least eight were collected. I don't necessarily care how many samples were collected outside of the one that was sequenced, but find the discrepancy odd.
5. It is in the methods stated that 10X genomics read cloud DNA libraries were, generated, but these sequences are nowhere to be seen either in the data repository, nor do they appear to have been used for the assembly, were these in fact generated or not?
6. It is stated that the genome was assembled with hifi-asm followed by purge_dups. Does this mean that you a) disabled the purge_dups step that is included in hifi-asm and ran it separately? b) purged duplications both with the built in and with the standalone  c) only ran it with the built in one? This question would likely have been answered if the methods were more clearly stated or if scripts were supplied.
7. I only have some experience working with Hi-C data and interpreting the contact maps, but Ï must say that I find the one presented confusing, especially as the caption states that chromosomes can be seen in decreasing order of size. Compared to other DTOL genomes this contact map is very unclear and I am unable to identify chromosome boundaries within it. There also appear to be quite a lot of trans connections that makes me wonder if this is the contact map that was generated before or after fixing miss-assemblies.
8. Lastly, I am a bit curious and concerned how the QV metric was estimated. Assuming it refers to basecall accuracy of the assembly on a phred scale, a number of 66 should mean that you have less than one base error per 1MB, and therefor less than 500base errors in the entire assembly. I have a hard time understanding how a non T2T, non-busco complete and unphased assembly can have a QV score that high. It might however be possible that I am not understanding how the QV score was calculated in this case, but in that case I think it would be nice if you could provide an explanation to how it was done.

Are sufficient details of methods and materials provided to allow replication by others?

Partly

Is the rationale for creating the dataset(s) clearly described?

Yes

Are the datasets clearly presented in a useable and accessible format?

No

Are the protocols appropriate and is the work technically sound?

Partly

Reviewer Expertise:

Evolutionary genetics, comparative genomics and genome assembly.

I confirm that I have read this submission and believe that I have an appropriate level of expertise to state that I do not consider it to be of an acceptable scientific standard, for reasons outlined above.

# Response

**Published:** 2025-02-15

**Authors:** Tree of Life Team Sanger

**Affiliations:** Wellcome Trust Sanger Institute, UK

Thank you for the helpful review.

 We have added explanatory text about the MerquryFK method for obtaining the QV statistic. This is not an uncommon score for PacBio HiFi read data - the base-error measurement QV was 66.4 as assessed before assembly in GenomeScope and after assembly, assessed using MerquryFK. We have also made other changes to the data note which are detailed in the Version comments.
